# Incidental parathyroidectomy during total thyroidectomy and functional parathyroid preservation: a retrospective cohort study

**DOI:** 10.1186/s12893-023-02176-3

**Published:** 2023-09-06

**Authors:** Charlotte Melot, Gabrielle Deniziaut, Fabrice Menegaux, Nathalie Chereau

**Affiliations:** 1 Department of General and Endocrine Surgery, Pitié Salpêtrière Hospital, APHP, Sorbonne University, 47-83 Boulevard de L’Hôpital, Paris, 75013 France; 2 Department of Pathology, Pitié Salpêtrière Hospital, APHP, Sorbonne University, Paris, France; 3 Groupe de Recherche Clinique N°16 Thyroid Tumors, Sorbonne University, Paris, France

**Keywords:** Total thyroidectomy, Parathyroid gland, Incidental parathyroidectomy, Parathyroid hormone, Hypocalcemia, Hypoparathyroidism

## Abstract

**Background:**

The published rate of incidental parathyroidectomy (IP) during thyroid surgery varies between 5.8% and 29%. The risk factors and clinical significance of postoperative transient hypocalcemia and permanent hypoparathyroidism are still debated. The aims of this study were to assess the clinical relevance of avoidable IP for transient hypocalcemia and permanent hypoparathyroidism, and to describe the risk factors for IP.

**Methods:**

This retrospective cohort study included 1,537 patients who had a one-step total thyroidectomy in a high-volume endocrine surgery center between 2018 and 2019. Pathology reports were reviewed for incidentally removed parathyroid glands. Intrathyroidal parathyroid glands were excluded from the study. Demographic characteristics, potential risk factors, and postoperative calcium and PTH levels were compared between IP and control groups.

**Results:**

Avoidable IP occurred in 234 (15.2%) patients. Patients with IP had a higher risk of transient hypocalcemia (17.9% vs. 11.5%, *p* = 0.006; odds ratio [OR] 1.68, 95% confidence interval [95% CI]1.16–2.45) and permanent hypoparathyroidism (4.7% vs. 1.6%, *p* = 0.002; OR 3.01, 95% CI 1.29–6.63) than patients without IP. Multivariate analysis showed that central lymph node dissection (CLND) and incidental removal of thymus tissue were independent risk factors for IP (OR 4.83, 95% CI 2.71–8.86, *p* < 0.001 and OR 1.72, 95% CI 1.02–2.82, *p* = 0.038).

**Conclusions:**

Patients with IP were more likely to develop transient hypocalcemia and permanent hypoparathyroidism, indicating the clinical significance of avoidable IP for patients and the need for raising awareness among surgeons. Patients undergoing CLND are at a higher risk for IP, and should be adequately informed and treated. Any removal of thymus tissue should be avoided during CLND.

## Introduction

The parathyroid glands finely regulate the calcium level through parathormone secretion. They are most usually located beside, or less commonly inside, the thyroid gland.

Thyroid surgery increases the risk of causing harm to the parathyroid glands by devascularizing or accidentally resecting them (incidental parathyroidectomy [IP]). Although the trend is toward reducing the extent of surgical management [1, 2], bilateral surgical procedures are still indicated in case of aggressive thyroid malignancy, multinodular goiters with compression symptoms, or some hyperthyroidism such as Graves’ disease [3, 4].

Postoperative hypocalcemia is the most common complication of bilateral thyroid surgery [5–7]. Transient hypocalcemia may lead to prolonged hospital stay [8] and increased medical costs. Permanent hypoparathyroidism may cause severe clinical issues (e.g., cataracts, nephrolithiasis) and considerable impairment of the quality of life of patients [9, 10].

The effect of IP on postoperative transient hypocalcemia and permanent hypoparathyroidism is still debated, mainly because the appropriateness of the studied populations was sometimes questionable (e.g., inclusion of lobectomy cases and insufficient population size). Identifying the risk factors for IP may enable exercising appropriate caution in at-risk patients. In previous studies describing IP, the reported incidence of intrathyroidal parathyroid glands on pathology reports varied between 0.2% and 10.2% [11, 12]. As all previous studies included intrathyroidal parathyroid glands in their analysis, they did not distinguish between avoidable and non-avoidable IP.

The aims of this study were to assess the clinical significance of avoidable IP for postoperative transient hypocalcemia and permanent hypoparathyroidism, and to identify the risk factors for avoidable IP in order to help reduce its occurrence.

## Methods

### Study population

Consecutive patients with benign or malignant thyroid disorders who underwent total thyroidectomy (in one-step) performed at a single tertiary center from January 2018 to December 2019 were enrolled in the study. Data were retrieved from a maintained institutional database, whose creation was approved by Institutional Research Ethics Committee (Ethic Committee of Paris region VI in Pitié Salpêtrière Hospital, registration number 20200115171338). According to the French law, retrospective studies built exclusively on data, do not need the approval of "committees for the protection of individuals”.

The main indications for surgery were multinodular compressive goiters, malignant or suspicious nodules on fine-needle aspiration biopsy, Grave’s disease, and multinodular goiters with hyperthyroidism.

**Fig. 1:**
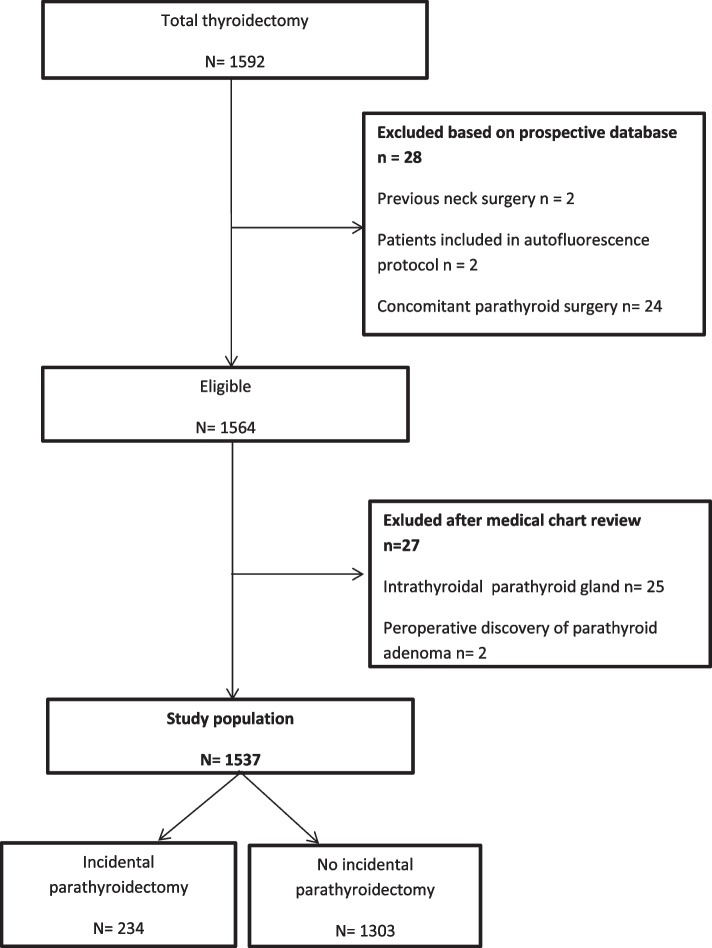
Flow chart

The exclusion criteria from the prospective database were previous neck surgery, concomitant parathyroid surgery, and inclusion in an autofluorescence study. The exclusion criteria after reviewing the operative and pathology reports were intentionally removed parathyroid glands (perioperative discovery of parathyroid adenoma). Cases of intrathyroidal IP were also excluded to keep the study focus on avoidable IP (Fig. 1).

Most of the data were prospectively collected from our institutional database. Demographic characteristics included: age, sex, body mass index, and medical history. Operative characteristics included lymph node dissection (LND) and autotransplantation of parathyroid glands. Pathologic characteristics included the size and weight of the thyroid and the final thyroid pathology. We reviewed all pathology reports for the presence, number, and location of the resected parathyroid glands; associated chronic thyroiditis; and associated incidentally resected thymus.

### Surgical technique

All surgeries were performed in a similar fashion by 7 endocrine surgeons. Regarding surgeon’s volume, each surgeon in our specialized department performed at least 100 endocrine cervical procedures per year.

Extracapsular dissection was systematically performed. The recurrent laryngeal nerve (RLN) was mandatorily identified. Intraoperative recurrent nerve monitoring was limited to large goiters and thyroid carcinoma with central lymph node metastasis. Attempts to identify the inferior parathyroid glands were not systematically made to avoid excessive dissection. Devascularized parathyroids were maintained in situ, whereas accidentally removed glands were autotransplanted in the ipsilateral sternocleidomastoid muscle. For autotransplantation, the parathyroid glands were divided into ≤ 1 mm pieces and slid into a muscular pouch. Suction drainage was never used. We did not perform any subtotal thyroidectomy.

Prophylactic level VI LND (central LND [CLND]) was performed for all thyroid cancers diagnosed on preoperative ultrasound-guided fine-needle aspiration cytology (Bethesda VI), or on intraoperative frozen-section analysis with a clinically node-negative (N0) result. Therapeutic LND of the invaded compartment was performed for cytologically proven or echographically suspected lymph node metastases. This approach is based on French Recommendations and studies’ demonstrating that prophylactic CLND permits an accurate staging of the disease that may guide subsequent treatment and follow-up.

### Pathologic findings

All pathologic examinations of thyroid specimens were performed by 17 experienced pathologists at the institution. “Intrathyroidal parathyroid” was defined as being totally surrounded by the thyroid.

### Postoperative follow-up

Serum total calcium levels were measured on postoperative day 1 and repeated on postoperative day 2 if the postoperative day 1 result was < 8 mg/dL. Plasma parathyroid hormone levels were measured 20 min after the completion of the thyroidectomy. Hypocalcemia was defined as a serum calcium level of < 8 mg/dL on at least 1 postoperative measurement. Symptomatic hypocalcemia could not be assessed because of lack of data. Hypoparathyroidism was considered permanent if oral calcium supplements were required after 6 months with plasma parathyroid hormone levels of < 15 pg/mL.

The protocol for treatment of postoperative hypocalcemia was oral calcium (1–4 g oral calcium carbonate daily) with vitamin D (alfacalcidol 0.5–1 µg daily) supplementation if the calcium level was < 7.2 mg/dL for 3 to 6 weeks. All patients were evaluated at the outpatient clinic 6 weeks after surgery with blood tests (calcium and plasma parathyroid hormone levels). Patients with persistent hypocalcemia were evaluated at 3, 6, and 12 months by their surgeon through blood testing.

### Statistical analysis

Our database was prospectively maintained. Statistical analyses were performed using R software. The normal distribution of quantitative variables was assessed using histograms. Proportions were compared using the chi-squares test or Fisher’s exact test, as appropriate. Student’s t-test or analysis of variance was used for quantitative variables. Logistic regression analysis was performed. Values are expressed as mean ± standard deviation. Statistical significance was set at *p* < 0.05.

## Results

### Clinical characteristics

As seen on Fig. 1, 1592 consecutive patients underwent one-step total thyroidectomy between January 2018 and December 2019. A total of 28 patients were excluded based on prospective database including 24 concomitant parathyroid surgery. Among the 1564 remaining eligible patients, 27 were exclude after medical chart review, including 25 for intrathyroidal parathyroid gland removal.

A total of 1537 total thyroidectomies were included (1187 [77.2%] women, median age: 51.9 years (range: 11.8–92.7 years)). Concomitant CLND was performed in 472 cases (30.7%).

A total of 569 (37%) cases of thyroid malignancy were found in pathologic examination, including 497 papillary carcinoma (87.3%), 32 follicular carcinoma (5.6%), 24 medullary carcinoma (4.2%), 8 papillary with follicular carcinoma (1.1%), 3 papillary with medullary carcinoma (0.5%), and 5 other carcinomas (0.9%).

A total of 266 parathyroid glands were incidentally removed in 234 patients, yielding an IP rate of 15.2%. A total of 203 patients had 1 gland removed (76.3%), 30 patients had 2 glands removed (11.3%) and 1 patient had 3 glands removed (0.4%). In addition, 57 patients had small fragments of parathyroid tissue found in pathologic specimens (3.7%). Of the removed glands, 194 were from beside the thyroid (73%) and 72 were from the central neck dissection (27%). 107 were removed from the right side and 106 glands from the left side, whereas 53 had no identified location in the pathology report. With respect to histologic characteristics, only 6 glands were diagnosed with adenoma (2.7%) and 2 were hyperplasic glands (0.7%).

Forty-eight patients had parathyroid autotransplantation in the ipsilateral sternocleidomastoid muscle including 1 patient with 2 parathyroid glands autotransplanted (3.1%).

### Relation between IP, hypocalcemia and hypoparathyroidism

The biochemical and clinical outcomes of patients with and without IP were analyzed and are summarized in Table 1. The rates of biochemical hypocalcemia on postoperative day 1 and the parathormone level were significantly different between the 2 groups. Biochemical hypocalcemia at postoperative day 1 occurred in 17.1% of patients with IP and 9.6% of patients without IP (OR 1.94, 95% CI 1.31–2.8, *p* < 0.001). The prevalence of postoperative transient hypocalcemia was 12.5% (17.9% when IP occurred versus 11.5% when it did not; OR 1.68, 95% CI 1.16–2.45, *p* = 0.006). The permanent hypoparathyroidism rate was 2.1%. Patients with IP had a significantly higher risk of developing permanent hypoparathyroidism than those without IP (4.7% vs. 1.6%, OR 3.01, 95% CI 1.29–6.63, *p* = 0.002).

**Table 1:** Influence of incidental parathyroidectomy on the risk of transient hypocalcemia and permanent hypoparathyroidism. Serum calcium and parathyroid hormone levels after total thyroidectomy

	**N (*n* = 1537)**	**IP *n* = 234**	**No IP *n* = 1303**	***P* value**	**OR (95% CI)**
**Serum calcium level at POD1 (mg/dL)**^a^	8.74 ± 0.8	8.62 ± 0.8	8.78 ± 0.4	< 0.001	
**Parathormone level at 20 min (pg/mL)**^**a**^	24.2 ± 19.9	17.6 ± 15	25.4 ± 20.5	< 0.001	
**Biochemical hypocalcemia at POD1** ƚ	165 (10.8%)	40 (17.1%)	125 (9.6%)	< 0.001	1.94 (1.31–2.85)
**Transient hypocalcemia**	192 (12.5%)	42 (17.9%)	150 (11.5%)	0.006	1.68 (1.16–2.45)
**Permanent hypoparathyroidism**	32 (2.1%)	11 (4.7%)	21 (1.6%)	0.002	3.01 (1.29–6.63)

*POD1* Postoperative day 1, *IP* Incidental parathyroidectomy

^a^values are mean (s.d.)

^b^serum calcium level < 8 mg/dL

With respect to transient hypocalcemia and permanent hypoparathyroidism, significant differences were found depending on the number of resected parathyroid glands (Table 2).

**Table 2:** Influence of number of resected parathyroid glands on transient and permanent hypocalcemia

	**No IP (*n* = 1303)**	**1 PG removed (*n* = 203)**	**2 PG removed (*n* = 30)**	***P* value**
**Transient hypocalcemia**	150 (11.5%)	38 (18.7%)	4 (13.3%)	0.029
**Permanent hypoparathyroidism**	21 (1.6%)	7 (3.4%)	4 (13.3%)	0.002
**Serum calcium level at POD1 (mg/dL)**	8.78 ± 0.6	8.62 ± 0.6	8.62 ± 0.8	< 0.001
**PTH at 20 min (pg/mL)**	25.4 ± 20	17.9 ± 15	15.9 ± 15	< 0.001

*IP* Incidental parathyroidectomy, *PG* Parathyroid gland, *POD1* Postoperative day 1, *PTH* Parathyroid hormone level

### Risk factors of incidental parathyroidectomy

On univariate analysis, IP was significantly associated with thyroid gland weight (*p* < 0.0001), malignant disease (*p* < 0.001), Grave’s disease (*p* = 0.001), CLND (*p* < 0.001), chronic thyroiditis (*p* = 0.004) and incidentally removal of thymus tissue (*p* < 0.001). A thyroid weight > 30 g (*p* < 0.001) and Grave’s disease (*p* = 0.001) seemed to be protective factors for IP. The multivariate analysis showed only CLND and incidental thymus resection as independent risk factors for IP (OR 4.83, 95% CI 2.71–8.86, *p* < 0.001 and OR 1.72, 95% CI 1.02–2.82, *p* = 0.0381, respectively). These results are summarized in Table 3.

**Table 3:** Univariate and multivariate analysis to identify risk factors for avoidable incidental parathyroidectomy

	**IP (*n* = 234)**	**No IP (*n* = 1303)**	**Univariate analysis *p* value**	**Multivariate analysis OR (IC 95%)**
Sex, male	52 (22.2%)	298 (22.9%)	0.828	
Age^a^	51.5 ± 15.4	51.1 ± 14.9	0.716	
BMI^a^	26.5 ± 5.5	26.4 ± 5.2	0.865	
Thyroid weight^a^	38.6 ± 42	56.7 ± 57	< 0.001	
Malignant	143 (61.1%)	426 (32.7%)	< 0.001	
**Grave’s disease**	**29 (12.4%)**	**284 (21.8%)**	**0.001**	
**CLN dissection**	**143 (61.1%)**	**329 (25.3%)**	**< 0.001**	**4.83 (2.71–8.86)**
CLN invasion	55 (42.3%)	128 (42.5%)	0.966	
**Chronic thyroiditis**	**89 (38%)**	**373 (28.7%)**	**0.004**	
**Thymus tissue**	**29 (12.4%)**	**51 (3.9%)**	**< 0.001**	**1.72 (1.02–2.82)**

*CLN* Central lymph node, *BMI* Body mass index, *IP* Incidental parathyroidectomy

^a^values are mean (s.d.)

### CLND

In the 472 cases of CLND, 72 cases of IP were found in 66 patients. Of them, 60 patients had 1 resected gland, 5 patients had 2 resected glands, and 1 patient had 3 resected glands. A total of 70 CLND specimens contained thymus tissue (14.8%). An association was found between the presence of incidentally resected thymus tissue in CLND specimens and IP (odds ratio [OR] 1.79; 95% confidence interval [CI] 1.06–3.02, *p* = 0.028). In addition, the mean number of lymph nodes found in CLND also did not differ between patients with and without incidental thymus resection (5.1 ± 4.6 vs. 5.5 ± 7.8, *p* = 0.579). The mean number of positive lymph nodes in CLND also did not differ between patients with and without incidental thymus resection (1.5 ± 3.1 vs. 1.3 ± 2.4, *p* = 0.432). These results are summarized in Table 4.

**Table 4:** Influence of unintentional thymus resection on incidental parathyroidectomy, positive and total numbers of lymph node in patients undergoing central lymph node dissection during total thyroidectomy for thyroid malignancy

	***N* = 472**	**Thymus tissue (*n* = 70)**	**No thymus tissue (*n* = 402)**	***P* value**	**OR (95% CI)**
**Incidental parathyroidectomy**	143 (30.3%)	29 (41.4%)	114 (28.4%)	0.0281	1.79 (1.06–3.02)
**Total number of LN**^a^	5.4 ± 6.9	5.1 ± 4.6	5.5 ± 7.8	0.579	
**Positive LN**^a^	1.4 ± 2.6	1.5 ± 3.1	1.3 ± 2.4	0.432	

*LN* lymph node

^a^values are mean (s.d.)

## Discussion

IP is a common finding in pathology reports of thyroid surgeries. Previous series have described IP rates between 5.8% and 29% [13, 14]. The wide range of IP rates may be explained by the different types of thyroid surgery described in those series (total thyroidectomy with or without CLND, subtotal thyroidectomy, lobectomy, isthmusectomy). In studies that focused on total thyroidectomy, the IP rate varied between 16.2% and 22.4% [15, 16]. Our study found an avoidable IP rate (excluding intrathyroidal IP) of 15.2%, which are consistent with recent literature reports.

The incidence of intrathyroidal parathyroid glands in cadaver studies is always very low [17, 18]. However, the reported incidence of intrathyroidal parathyroid glands on pathology reports of previous studies describing IP varied between 0.2% and 10.2% of all thyroid specimens [11, 12], representing between 2.2% and 57.5% of resected parathyroid glands. We chose to exclude intrathyroidal parathyroid glands to keep the study focus on avoidable IP.

In our study, the incidence of transient hypocalcemia was significantly higher in patients with IP. The association between IP and transient hypocalcemia remains debated, because many studies did not find any statistical link between IP and transient hypocalcemia. However, the primary objective of those studies was to provide a description of IP, and they included patients undergoing less than total thyroidectomy or a small number of total patients [19–21]. Recent reports that focused on patients undergoing total thyroidectomy or total thyroidectomy with central neck dissection, mainly found a link between IP and transient hypocalcemia [16, 22, 23].

In our study, patients who had IP were more likely to present with permanent hypoparathyroidism, even those with only 1 incidentally removed parathyroid gland. A 3-fold increased risk of permanent hypoparathyroidism was found in patients with IP after total thyroidectomy. The influence of IP on permanent hypoparathyroidism remains controversial. An association between permanent hypoparathyroidism and IP has been reported in only a few studies [11, 14, 22–25]. This may be explained by the low incidence of permanent hypoparathyroidism, or the moderate incidence of IP associated with the insufficient sample size of many studies. Studies reporting an association between permanent hypoparathyroidism and IP had a rather large population [23], an elevated IP rate [14, 22, 25] or an elevated permanent hypoparathyroidism rate [11, 22, 24].

Many studies have attempted to identify the potential risk factors for IP.

Demographic data such as young age and female sex have been reported as risk factors for IP [12, 26, 27]. However, this was concluded on univariate analysis and was not verified using multivariate models. In our study, neither age nor sex was different between patients with and without IP.

With respect to the final pathology report, many studies have reported malignancy as a risk factor for IP [16, 23]. In our study, the incidence of malignancy was significantly higher in patients with IP on univariate analysis. However, on multivariate analysis, malignancy was not an independent risk factor for IP. We considered that it may be a confounding factor because a large majority of our patients with malignancy had CLND.

Some studies have reported Grave’s disease, chronic thyroiditis, and thyroid weight as risk factors for IP [11, 28–30]. In contrast, in our study, the incidence of Grave’s disease and thyroid weight was significantly lower in patients with IP on univariate analysis, although they were not independent protective factors in multivariate analysis. For Grave’s disease (small thyroid gland in most cases), this result was consistent with at least 1 other published study with a large sample size [11]. We hypothesized that as we expected a more difficult dissection and a higher rate of postoperative hypocalcemia in those patients, we actually were more careful about parathyroid gland preservation. On the opposite, we found that IP was more frequent in case of Hashimoto thyroiditis, but not in the multivariate model. We assumed that thyroiditis was involved in postoperative hypocalcemia, but not by increasing the IP rate.

Many previous studies have reported CLND as a risk factor for IP. In our study, CLND was the strongest independent risk factor for IP, with a ≥  4-fold increased risk of IP in patients with CLND. Therefore, surgeons should be aware of the risk–benefit ratio of CLND for each patient and patients should be adequately informed. We found that if some unintentionally resected thymus tissues were recorded in the central neck dissection in the pathology report, the risk of IP significantly increased. To our knowledge, this is the first study to report the incidental resection of thymus tissue as an independent risk factor for IP. Moreover, the presence of thymus tissue in CLND specimens did not improve the quality of the CLND because the number of resected and positive lymph nodes was the same in the 2 groups. We suggest avoiding resecting any thymus tissue during central neck dissection. Some authors have developed a surgical technique to preserve the thymus during central neck dissection with a significantly improved rate of inferior parathyroid preservation [31].

### Strength and limitations

The strengths of our study were that despite its retrospective nature, patient data were recorded consecutively and prospectively with great thoroughness. Calcium and parathormone blood levels were systematically collected and assessed to ensure that all cases of transient and permanent hypocalcemia were recorded. The long-term follow-up allowed us to determine the long-term consequences of IP.

However, the present study had some limitations. Most surgeons did not document the number of identified parathyroid glands or the macroscopic vascularization, precluding any correlation analysis between parathyroid identification and IP. We believe that parathyroid glands do not need to be routinely identified and assessed if they are not encountered during thyroidectomy [32].

We excluded patients whose pathology reports mentioned only “parathyroid tissue” to include only those who had 1 or several whole gland(s) incidentally removed. Because of the variability of pathologists who evaluated the specimens and the quasi-constant lack of size measurements of the resected “parathyroid tissue”, we could have potentially excluded a small number of patients with IP.

## Conclusion

Avoidable incidental parathyroidectomy has a rather high incidence and has clinical significance for transient hypocalcemia and permanent hypoparathyroidism. Central lymph node dissection and incidental thymus tissue resection are independent risk factors for IP. Removal of thymus tissue should be avoided during central neck dissection, and the indications for CLND should be carefully discussed for individualized care.

## Data Availability

The dataset analyzed during the current study are available from the corresponding author upon reasonable request (charlotte.melot@aphp.fr).
